# The Effect of Congruent Tibial Inserts in Total Knee Arthroplasty: A Network Meta-Analysis of Randomized Controlled Trials

**DOI:** 10.3390/life13091942

**Published:** 2023-09-21

**Authors:** Yen-Lin Tsai, Sung Huang Laurent Tsai, Chia-Han Lin, Chun-Ru Lin, Chih-Chien Hu

**Affiliations:** 1 Department of Orthopedic Surgery, Chang Gung Memorial Hospital, Linkou Medical Center, No. 5, Fu-Hsin St. Kweishan County, Taoyuan 333, Taiwan; 2 Bone and Joint Research Center, Chang Gung Memorial Hospital, Linkou Medical Center, No. 5, Fu-Hsin St. Kweishan County, Taoyuan 333, Taiwan; 3 Department of Orthopaedic Surgery, Chang Gung Memorial Hospital, Keelung Branch, Keelung 204, Taiwan; 4 Department of Medicine, MacKay Medical College, New Taipei City 252, Taiwan; 5 College of Medicine, Chang Gung University, Taoyuan 333, Taiwan

**Keywords:** ultracongruent, medial congruent, total knee arthroplasty, meta-analysis, outcomes

## Abstract

**Objective**: The aim of this study was to determine whether modern congruent tibial inserts are associated with superior outcomes in total knee arthroplasty (TKA). **Background**: Ultracongruent fixed-bearing (UCFB) and medial congruent fixed-bearing (MCFB) inserts have been known to be effective in total knee arthroplasty with patient satisfaction. Nonetheless, no supporting evidence to date exists to rank the clinical outcomes of these various congruent inserts in TKA compared with other important considerations in TKA including cruciate-retaining fixed-bearing (CRFB) and posterior-stabilized fixed-bearing (PSFB) inserts. **Methods**: We searched PubMed, Embase, the Cochrane Central Register of Controlled Trials, Web of Science, and Scopus up to 15 May 2022. We selected studies involving an active comparison of UCFB or MCFB in TKAs. We performed a network meta-analysis (NMA) of randomized controlled trials (RCTs) and compared different congruent inserts. We ranked the clinical outcomes by SUCRA score with the estimate of the best treatment probability. Our primary outcomes were revision rates and radiolucent lines. Secondary outcomes were functional scores, including the range of motion (ROM), the Knee Society Score (KSS), the Oxford Knee Score (OKS), and WOMAC. **Results**: Eighteen RCTs with 1793 participants were analyzed. Our NMA ranked MCFB, CRFB, and UCFB with the lowest revision rates. CRFB and UCFB had the fewest radiolucent lines. UCFB had overall the best ROM. UCFB and MCFB had the best OKS score overall. **Conclusions**: The ranking probability for better clinical outcomes in congruent inserts demonstrated the superiority of congruent tibial inserts, including UCFB and MCFB. UCFB may be associated with better ROM and postoperative functional outcomes. However, integrating future RCTs for high-level evidence is necessary to confirm these findings.

## 1. Introduction

Bearing designs for total knee arthroplasty (TKA) have evolved over time to improve clinical outcomes. Traditional designs include posterior-stabilized (PS) and cruciate-retaining (CR) fixed-bearing and mobile-bearing designs. The posterior-stabilized (PS) design involves removing the anterior cruciate ligament (ACL) and replacing it with artificial components that provide posterior stability during knee motion. While the design offers advantages, such as enhanced posterior stability, it has drawbacks including non-physiological motion patterns due to ACL absence, potential restrictions in certain activities, and challenges in achieving the optimal surgical technique. Cruciate-retaining (CR) TKA is a surgical procedure that maintains the anterior cruciate ligament (ACL) while replacing damaged knee joint components with artificial implants. This design aims to preserve natural knee motion. However, drawbacks include potential limitations in certain activities due to ACL preservation, the risk of ACL degeneration over time, and the need for precise surgical skills to maintain ACL integrity. Patient suitability varies based on ACL health and activity needs, with some risk of posterior cruciate ligament (PCL) issues. Fixed-bearing designs provide rigid fixation of polyethylene inserts within the tibial implant [1], while mobile-bearing designs were developed to improve conformity and lower contact stresses with the goal of pursuing native knee kinematics [2,3,4]. However, complications such as bearing dislocations have been reported for mobile-bearing designs, while high contact stresses with polyethylene wear were reported for PS designs. A recent meta-analysis including revision rates, aseptic loosening, radiographic loosening/osteolysis, and functional outcomes has not proven one superior to another [1,3,5,6,7,8].

Ultracongruent (UC) and medial congruent (MC) fixed-bearing designs have been developed to improve the clinical outcomes of TKA in the past few decades. The UC design has a higher anterior lip of the insert instead of a post-cam mechanism to control posterior translation of the tibia in flexion after sacrificing the posterior cruciate ligament (PCL), while the MC design provides greater conformity of the medial compartment [9,10,11,12].

Ultracongruent (UC) fixed-bearing designs offer enhanced stability, reduced wear, optimized motion range, and minimized soft tissue issues. However, the complexity of their design, surgical expertise required, potential for component wear, patient suitability assessment, and cost implications raise concerns. On the other hand, medial congruent (MC) designs provide stability and wear reduction. Yet, they come with limitations on mobility, potential wear-related problems, stress distribution issues, alignment accuracy challenges, and variability in patient suitability. Both designs aim to avert post-cam wear and fractures while preserving femoral condyle notch bone. However, these potential benefits are balanced by raised concerns.

Some studies have suggested that UCFB designs may be associated with a decreased range of motion (ROM) compared to traditional posterior stabilized fixed-bearing (PSFB) designs [13,14,15,16]. Additionally, some studies have raised concerns about the potential of early loosening due to the restriction of the rotational freedom of the femur and increased stress on the tibial bone surface [17,18,19]. Despite these concerns, UCFB designs have been adopted by the manufacturers of most of the major prosthetic knee systems, such as the Persona knee system (Zimmer-Biomet), E-motion/Columbus (Aesculap), Genesis II (Smith and Nephew), and AMK (Depuy Synthes Model of the Anatomic Modular Knee).

Given the conflicting results and concerns regarding UCFB designs, it is important to determine if these designs are associated with superior/inferior outcomes in current TKA. The objective of this study was to conduct a network meta-analysis of randomized controlled trials to compare the clinical outcomes of various congruent tibial inserts in TKA, including UCFB designs, and determine if they are associated with different outcomes.

## 2. Methods

### 2.1. Research Protocol and Search Question

The research protocol for this study was developed to answer the following PICO question: In patients undergoing total knee arthroplasty (TKA), do congruent tibial inserts containing UC and MC designs result in lower revision rates, fewer radiolucent lines, and better clinical outcomes compared to traditional tibial inserts including CRFB and PSFB? The search protocol was based on the guidelines of the Preferred Reporting Items for Systematic Reviews and Meta-Analyses (PRISMA) statement and was registered with PROSPERO in January 2020. Only randomized controlled trials were included in the analysis. The present study did not involve individual patient data, and informed consent of participants was not applicable.

### 2.2. Eligibility Criteria and Primary Outcome

To be eligible for inclusion in this study, studies had to meet the following criteria: (1) included patients who underwent total knee arthroplasty with various congruent tibial inserts; (2) clearly defined the congruency of the insert used; (3) randomized controlled trials study published in the English language up to 15 May 2022; (4) reported the primary outcome of interest: revision rates and radiolucent lines. Secondary outcomes of interest included functional scores such as range of motion (ROM), the Knee Society Score (KSS), the Oxford Knee Score (OKS), and WOMAC. Studies were excluded if they met any of the following criteria: (1) were single-arm follow-up studies, case reports, case series, reviews, basic science experiments, or animal or cadaver studies; (2) included patients who underwent immunosuppression; (3) were conference abstracts.

### 2.3. Search Strategy and Study Selection

To identify relevant studies for inclusion in this study, we conducted a systematic search of multiple databases including PubMed, Embase, the Cochrane Central Register of Controlled Trials, Web of Science, and Scopus. The search was performed using a combination of keywords and medical subject headings (MeSH) that were adjusted for each database. The most recent search was conducted on 15 May 2022. Additionally, we also conducted a recursive search by reviewing the bibliographies of obtained articles.

We employed a two-step review process to evaluate the articles identified through the search. In the first step, two independent reviewers (YLT, SHLT) evaluated the titles and abstracts of the articles to determine their eligibility for inclusion in the study. In the second step, the full text of relevant articles was reviewed by the same two independent reviewers (YLT, SHLT) to assess their qualification for inclusion. Any disagreements between the reviewers were resolved through discussion.

### 2.4. Data Collection and Quality Assessment

The following data were extracted from each included study: author, year of publication, region of study, data source, study design, period of study, study arms, sample size, patient age, inclusion criteria, and the specific definition of each treatment arm.

Two independent reviewers (YLT, SHLT) assessed the risk of bias and overall quality of each study using the GRADE assessment tool. This tool was used to evaluate the confidence in effect estimates. Any discrepancies in the assessment of risk of bias or study quality were resolved through discussion between the reviewers.

### 2.5. Statistical Analysis and Quantitative Data Synthesis

A frequentist random-effects network meta-analysis (NMA) was conducted to compare the different congruent designs of TKA. This method allows for both direct and indirect comparisons among the included studies. Heterogeneity among the studies was evaluated using the τ2 statistic, with values of 0.04, 0.16, and 0.36 corresponding to low, moderate, and high degrees of heterogeneity, respectively.

To examine the potential for small-study bias such as publication bias, comparison-adjusted funnel plots and Egger tests were used. Subgroup analysis was performed based on treatment comparison to evaluate potential confounders. The probability of each treatment group being the best for the target outcomes was ranked using the surface under the cumulative ranking curve (SUCRA), which reflects the percentage of an intervention that was the best without uncertainty.

The potential inconsistency between the direct and indirect evidence in the network was evaluated using the loop-specific and node-splitting models. Additionally, a design-by-treatment interaction model was used to evaluate global inconsistency within the entire NMA.

## 3. Results

### 3.1. Literature Search and Selection Process

**Figure 1:**
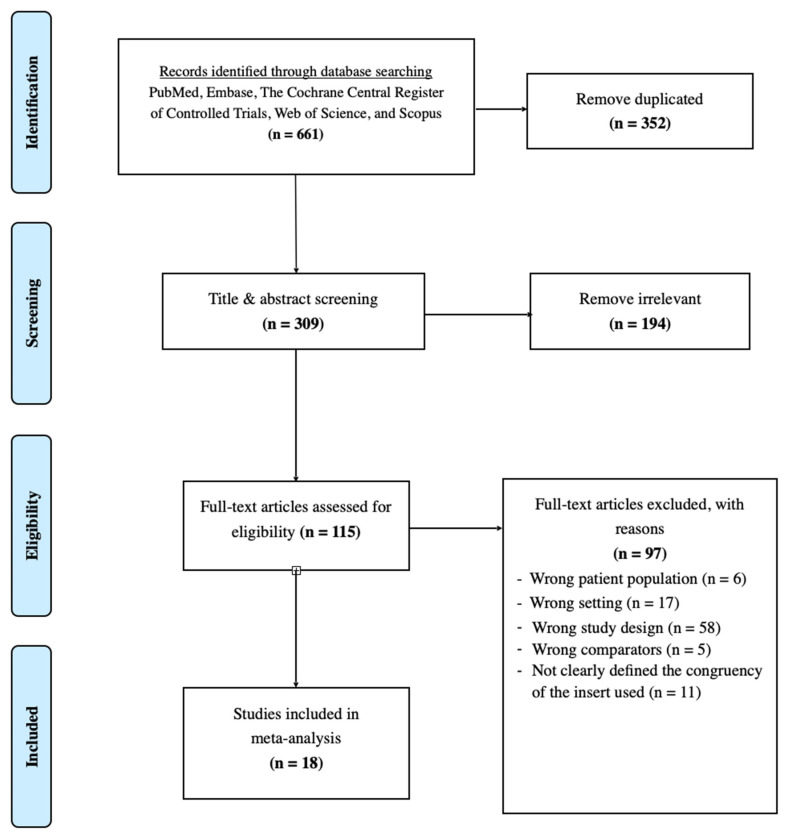
PRISMA flow diagram.

Our literature search identified a total of 661 articles through database searching. After removing duplicates and screening the titles and abstracts, 194 full-text articles were reviewed for eligibility. Ultimately, 18 studies were included in the network meta-analysis. The selection process is summarized in Figure 1.

### 3.2. Study Characteristics

A total of 18 RCT studies were included in the analysis. These studies included a total of 1793 patients. The mean patient age across studies ranged from 64.9 to 72.5 years; on average, 66.8% of patients were female (range 7% to 95%) (Table 1). The studies were conducted in various countries including China (one study, n = 46), Germany (two studies, n = 229), USA (four studies, n = 718), Korea (three studies, n = 250), India (two studies, n = 178), Australia (two studies, n = 165), Japan (two studies, n = 105), UK (one study, n = 80), and Sweden (one study, n = 22). All studies were active comparator studies, with various comparisons made between different congruent tibial inserts. The specific comparisons made are detailed in the provided text. Five articles compared ultracongruent fixed-bearing (UCFB) to standard posterior stabilized fixed-bearing (PSFB); two compared UCFB to CRFB; four compared MCFB to PSFB; one compared MCFB to PSRP; one compared MCFB to CRFB; one compared MCFB to CRFB and PSFB; one compared MCFB to PSFB; two compared MCFB to UCFB; and one compared MCFB to CRFB, PFFB, and PSRP.

### 3.3. Methodological Quality and Assessment of Risk of Bias

The methodological quality of the included studies was assessed using ROB2.0. Two studies were found to not provide sufficient follow-up protocol and length of follow-up for the outcome of interest. The strength of evidence for the outcome was based on the GRADE approach and was determined to be moderate. The network graph demonstrates the structure of our network meta-analysis, with the width of the edge corresponding to the number of studies.

### 3.4. Revision Rate

**Figure 2:**
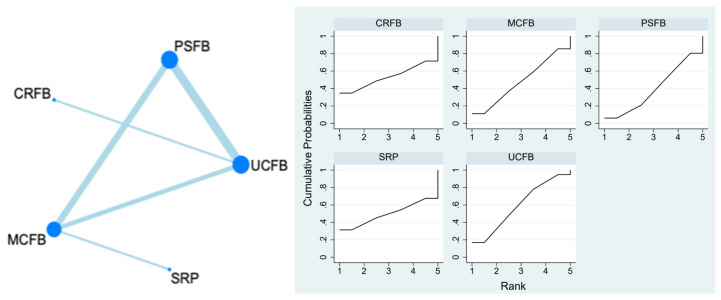
Revision rate.

This outcome was reported by 11 studies with 941 participants. CRFB (0/105, 0%), MCFB (0/260, 0%), and PSRP (0/92, 0%) had no revisions. UCFB had three revisions (3/364, 0.8%), and PSFB had five revisions (5/317, 1.6%). CRFB, MCFB, and UCFB were ranked the best among all the treatments for the outcome of revision rates, with the heterogeneity between groups not reaching statistical significance. The SUCRA plot is in Figure 2. The detailed numbers are presented in Appendix A(A).

A. The Network Plot of Revision Rates. In a network plot, each node represents a specific insert type, with its size proportional to the number of RCTs in the node. Connections between nodes represent direct comparative studies between insert types.
B. The SUCRA Plot of Revision Rates. The SUCRA plot illustrates the ranking probabilities of different interventions based on the surface under the cumulative ranking (SUCRA) curve. The *x*-axis represents the interventions or treatments being compared, while the *y*-axis represents the SUCRA values ranging from 0 to 100%. Higher SUCRA values indicate better treatment rankings. Each bar in the plot corresponds to a treatment, and its length represents the SUCRA value. The SUCRA plot provides a visual representation of the relative efficacy of different interventions, helping to identify the most effective treatment options in a given analysis.

### 3.5. Radiolucent Lines

**Figure 3:**
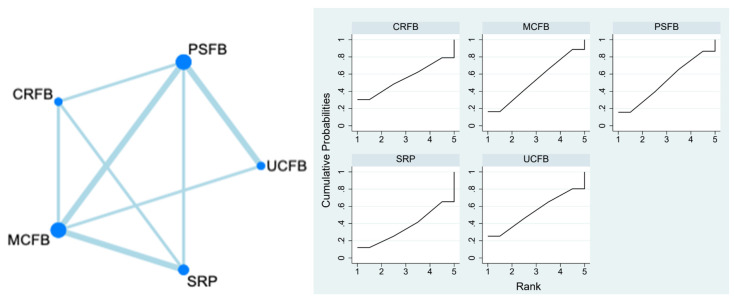
Radiolucent lines.

This outcome was reported by five studies (Kim, Uvehammer, Indelli, Ishida, Pricchett, and Hossain et al.) with 797 participants. No radiolucent lines were found in CRFB (0/215, 0%) and UCFB (0/86, 0%). Three radiolucent lines were found in PSFB (3/301, 1%), which was 2 years after surgery on average. Five radiolucent lines were found in PSRP (5/175, 2.9%), which was 2.6 years after surgery on average. Ten radiolucent lines were found in MCFB (10/401, 2.5%), which was 2.5 years after surgery on average. CRFB and UCFB were ranked the best among all the treatments for the outcome of radiolucent lines, with the heterogeneity between groups not reaching statistical significance. The SUCRA plot is in Figure 3. The detailed numbers are presented in Appendix A(B,H).

A. The Network Plot of Radiolucent Lines. In a network plot, each node represents a specific insert type, with its size proportional to the number of RCTs in the node. Connections between nodes represent direct comparative studies between insert types.
B. The SUCRA Plot of Radiolucent Lines. The SUCRA plot illustrates the ranking probabilities of different interventions based on the surface under the cumulative ranking (SUCRA) curve. The *x*-axis represents the interventions or treatments being compared, while the *y*-axis represents the SUCRA values ranging from 0 to 100%. Higher SUCRA values indicate better treatment rankings. Each bar in the plot corresponds to a treatment, and its length represents the SUCRA value. The SUCRA plot provides a visual representation of the relative efficacy of different interventions, helping to identify the most effective treatment options in a given analysis.

### 3.6. Range of Motion (ROM)

**Figure 4:**
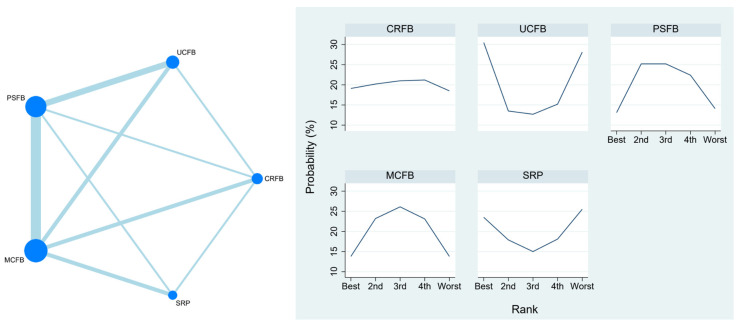
Range of motion.

This outcome was reported by 13 studies with 1651 participants. The mean ROM of UCFB was 111.63°. UCFB was ranked the best among all the treatments for the outcome of range of motion, with the heterogeneity between groups not reaching statistical significance. The SUCRA plot is in Figure 4. The detailed numbers are presented in Appendix A(C,H).

A. The Network Plot of Radiolucent Lines. In a network plot, each node represents a specific insert type, with its size proportional to the number of RCTs in the node. Connections between nodes represent direct comparative studies between insert types.
B. The SUCRA Plot of Radiolucent Lines. The SUCRA plot illustrates the ranking probabilities of different interventions based on the surface under the cumulative ranking (SUCRA) curve. The *x*-axis represents the interventions or treatments being compared, while the *y*-axis represents the SUCRA values ranging from 0 to 100%. Higher SUCRA values indicate better treatment rankings. Each bar in the plot corresponds to a treatment, and its length represents the SUCRA value. The SUCRA plot provides a visual representation of the relative efficacy of different interventions, helping to identify the most effective treatment options in a given analysis.

### 3.7. Aseptic Loosening

This outcome was reported only by four studies (Lutzner, Kim, Indelli, and Kulshrestha et al. [20,22,26,29]) with 369 participants. No aseptic loosening was found in MCFB (0/90, 0%). One aseptic loosening was found in UCFB (1/98, 1%), which was 29 months after surgery. For all the treatments, the heterogeneity between groups did not reach statistical significance. The detailed numbers are presented in Appendix A(H).

### 3.8. The Knee Society Score (KSS)

This outcome was reported by 15 studies with 1593 participants. The mean KSS of CRFB was 78.3. The mean KSS of UCFB was 86.11. The mean KSS of PSFB was 90.75. The mean KSS of MCFB was 88.44. The mean KSS of PSRP was 92. PSRP was ranked the best among all the treatments, with the heterogeneity between groups not reaching statistical significance. The detailed numbers are presented in Appendix A(D).

### 3.9. The Knee Society Score—Function (KSS-F)

This outcome was reported by 11 studies with 1232 participants. The mean KSS-F of CRFB was 68. The mean KSS-F of UCFB was 50.42. The mean KSS-F of PSFB was 61.71. The mean KSS-F of MCFB was 69.14. The mean KSS-F of PSRP was 83.55. PSRP was ranked the best among all the treatments, with the heterogeneity between groups not reaching statistical significance. The detailed numbers are presented in Appendix A(E).

### 3.10. The Knee Society Score—Pain (KSS-P)

This outcome was reported by four studies with 306 participants. The mean KSS of UCFB was 44.5. The mean KSS of PSFB was 46.5. The mean KSS of MCFB was 42.6. The mean KSS of PSRP was 45. PSFB was ranked the best among all the treatments, with the heterogeneity between groups not reaching statistical significance.

### 3.11. Oxford Knee Score (OKS)

This outcome was reported by seven studies with 636 participants. The mean OKS of CRFB was 37. The mean OKS of UCFB was 40.05. The mean OKS of PSFB was 36.22. The mean OKS of MCFB was 36.46. UCFB and MCFB were ranked the best among all the treatments, with the heterogeneity between groups not reaching statistical significance. The detailed numbers are presented in Appendix A(F).

### 3.12. WOMAC

This outcome was reported by seven studies with 561 participants. The mean WOMAC of CRFB was 20.8. The mean WOMAC of UCFB was 14.56. The mean WOMAC of PSFB was 18.58. The mean WOMAC of MCFB was 18.48. CRFB was ranked the best among all the treatments, with the heterogeneity between groups not reaching statistical significance. The detailed numbers are presented in Appendix A(G).

## 4. Discussion

Modern bearing designs for total knee arthroplasty (TKA) have undergone significant advancements to optimize clinical outcomes. These developments have encompassed traditional options such as posterior-stabilized (PS), cruciate-retaining (CR), and mobile-bearing designs [16,36]. Nonetheless, it is important to acknowledge that mobile-bearing designs may present potential complications, such as insert dislocations, whereas posterior-stabilized (PS) designs are associated with elevated contact stresses and increased polyethylene wear. In recent years, fixed-bearing designs, specifically ultracongruent (UC) and medial congruent (MC) inserts, have emerged as promising alternatives aimed at improving outcomes in TKA. UC inserts feature an elevated anterior lip, while MC inserts offer superior conformity to the medial compartment [10,11,37]. Our analysis highlights the significance of ROM as a critical parameter for evaluating clinical outcomes across different bearing types in total knee arthroplasty. Drawing from data sourced from 13 studies encompassing 1651 participants, we observed that UCFB emerged as the frontrunner among all treatments in terms of range of motion, underscoring its potential advantage in this domain. Our findings also indicated fewer radiolucent lines with UCFB compared to others.

Our findings resonate with several studies that also investigated the impact of UCFB on ROM. For example, Ishida et al. concluded that UCFB showed an improvement of 5 degrees compared to the MCFB, underscoring UCFB’s potential to enhance patient ROM [34]. Similarly, Song et al. reported a 2-degree enhancement in ROM for UCFB compared to CRFB, further accentuating its potential benefit [24]. Laskin et al. also contributed to this discourse, noting a 3-degree ROM improvement with UCFB when compared to PSFB [21]. A recent prospective, randomized, single-center Level 1 study conducted by Scott et al. aimed to compare clinical outcomes between PS and medial-stabilized (MS) devices implanted with kinematic alignment [12]. Their study found that at the minimum 2-year follow-up, the results demonstrated the superiority of the medial-stabilized device in terms of multiple clinical outcomes, including Knee Society scores, Forgotten Joint Score (FJS), and ROM. The maximum flexion at 2 years was 132 in the MS group and 124 in the PS group (*p* < 0.0001). Scott et al. suggest that the superior clinical results of the medial-stabilized (MS) knee in their trial may be due to the MS implant offering enhanced mid-flexion stability compared to the posterior-stabilized (PS) design [12]. This greater stability could potentially result in improved function, performance, and patient satisfaction [12]. These findings highlight the importance of implant design and alignment techniques in optimizing TKA outcomes, which aligns with the broader context of our research on congruent tibial inserts. Our study not only reinforces these individual observations but collectively suggests that UCFB might indeed lead to improved ROM outcomes in total knee arthroplasty when compared to other bearing types. The SUCRA plot in Figure 4 substantiates our findings, underscoring UCFB’s consistently superior performance in ROM across multiple studies. Remarkably, the absence of statistical significance in heterogeneity between groups adds to the credibility of the observed trend. Nonetheless, it is vital to contextualize these findings within the broader clinical landscape. Patient-specific characteristics, surgical techniques, and implant designs can all influence outcomes. Furthermore, our analysis is not without limitations, warranting further research with robust methodologies to establish the clinical relevance of these findings. In essence, our analysis underscores UCFB’s promising potential to enhance the range of motion in total knee arthroplasty. The convergence of results from various studies, including those by Ishida et al., Song et al., Laskin et al., and Scott et al., provides a strong foundation for this observation [12,21,24,34]. This insight contributes to a better understanding of the clinical implications of different bearing types and can serve as guidance for clinicians when tailoring decisions to individual patient needs.

The results of our analysis indicate that UC designs provide fewer radiolucent lines. Additionally, our analysis showed that congruent inserts may lead to improved functional scores, such as the Oxford Knee Score and WOMAC [25,38]. These findings may be attributed to the higher anterior lip exactly controlling posterior translation of the tibia in flexion after sacrificing PCL requiring posterior stability. This higher anterior lip design also provides benefits such as preventing wear and fracture after the procedure and reducing femoral bone loss when compared to PSFB or PSMB [22]. Concerns about early loosening of tibial components due to the restriction of rotational freedom of the femur with increased stress on the tibial bone surface were not sufficiently identified in the current study [14,39].

UCFB designs have been found to have fewer overall radiolucent lines compared to other TKA designs [38,40]. One possible explanation for this finding is that the increased surface area of contact redistributes the contact stress between the tibial insert and the femoral component in UC design. This increased contact area leads to improved stability and less wear/tear on the components [22]. Additionally, the UC design may reduce the potential micromotion between the tibial insert and the femoral component, which can lead to radiolucency [13]. Another possible explanation for fewer radiolucent lines observed in UC designs may be the improved accuracy of implant positioning and alignment achieved with these new designs, which can also contribute to improved stability and reduced wear.

Our findings are consistent with previous studies that have also reported similar revision rates and radiolucent lines among different types of TKA designs [41]. The better conformity and contact area between the femoral component and the tibial plateau with UCFB may help explain the reduced radiolucent lines. This may lead to improved load distribution and reduced micromotion at the bone–implant interface, which can help prevent loosening and improve implant survival. Moreover, the reduced incidence of PS-related complications such as patellar clunk syndrome and patellar fracture with UCFB may also contribute to the better clinical outcomes observed [22,42]. A recent meta-analysis conducted by Vishwanathan et al. aimed to compare the outcomes of anterior stabilized UC and standard CR inserts in fixed-bearing primary total knee arthroplasty [43]. The study included 14 studies (3 RCTs and 11 comparative case-cohort studies), comprising 9989 knees, and found that knee pain was better in patients that had standard CR inserts, and the quality of life was also better in patients that had standard CR inserts. However, there was a 72% lesser chance of revision TKA or change of insert for postoperative instability in knees that had been implanted with UC inserts. There was no difference in the other outcome measures.

Wenzel et al. conducted a meta-analysis comparing UC, PS, and CR tibial inserts in TKA and concluded that there were no clinical differences between these implant types at 2 years after surgery [44]. In our study, we conducted a comprehensive analysis that included four distinct tibial insert types: UCFB, MCFB, CRFB, and standard rotating platform (SRP) inserts. This crucial difference allowed us to provide a more detailed examination of these various insert designs in TKA. Additionally, Wenzel et al. combined MCFB with UC inserts in their analysis, potentially blurring the specific outcomes associated with MCFB. In contrast, our study treated MCFB as a distinct category and evaluated its performance alongside other insert types. This approach provided a more nuanced understanding of the clinical outcomes associated with MCFB inserts. Therefore, while both studies contribute valuable insights, our research offers a broader and more granular analysis of tibial insert designs in TKA, making it a comprehensive resource for surgeons and researchers in the field. Importantly, our network meta-analysis incorporates both direct and indirect evidence, allowing for a more thorough assessment of the relative clinical performance of these insert types. By considering a wider array of insert designs and leveraging indirect evidence, our study provides a more in-depth understanding of the clinical outcomes associated with different tibial inserts in TKA. As such, it serves as a valuable resource for clinicians and researchers seeking a comprehensive overview of the available evidence to inform clinical decisions and future research endeavors in the field of total knee arthroplasty.

Our study has some limitations that should be considered when interpreting the results. Firstly, despite our inclusion of a substantial number of RCTs, the restricted availability of studies comparing CR-type rotating platforms with UCFB constrains us from offering definitive conclusions about the relative efficacy and safety of CR-type rotating platforms in comparison to UCFB. To comprehensively address this, future research should encompass larger sample sizes and extended follow-up periods. Secondly, the relatively short follow-up duration in the studies we examined, mostly less than 5 years, might not sufficiently capture potential long-term complications or time-dependent adverse events in TKA. Prolonged patient monitoring is essential to holistically assess the safety and efficacy of these implants. Furthermore, a notable limitation of our analysis is its assumption that all observed trends can be directly attributed solely to the bearing. However, it is vital to acknowledge that the behavior of the bearing’s articulation and the forces applied to the polyethylene component are also contingent on the design of the femoral component. Some knees within our studied population featured femoral components with fixed or dynamic radii of curvature, yet we did not deeply delve into this factor, potentially introducing a certain level of bias into our analysis. Lastly, the inherent heterogeneity across the included studies, spanning diverse patient characteristics, surgical techniques, implant designs, and outcome measures, could have influenced our analysis outcomes and limited the applicability of findings across various patient groups and implant variations. Our focused attention on the varying congruent designs of tibial inserts in TKA of our study may have resulted in the oversight of other conceivable influential factors, including patient comorbidities, diverse surgical techniques, and the precise alignment and positioning of implants. Further studies that encompass these factors are imperative to achieve a comprehensive understanding of how congruent tibial inserts genuinely impact TKA outcomes.

Network meta-analysis (NMA) is a method used to compare multiple interventions in a single analysis. However, it is important to note that NMA has some limitations, such as reliance on indirect comparisons which can introduce uncertainty and bias. Additionally, NMA requires a large amount of data to be effective, and when data are limited, the results may not be as reliable. Our study found that UCFB may be associated with fewer radiolucent lines and overall better clinical outcomes compared to other congruent inserts [25]. Further studies with more data and longer follow-up periods are needed to confirm these findings.

## 5. Conclusions

In conclusion, our study suggests that ultracongruent fixed-bearing (UCFB) and medial congruent fixed-bearing (MCFB) inserts may be good options for total knee arthroplasty (TKA) due to their potential to improve range of motion and clinical outcomes. The advantage of UCFB and MCFB over other inserts may encourage orthopedic surgeons to consider this design in their surgical decision making. However, given that our findings are based on indirect comparisons and limited studies, caution should be exercised when interpreting the results. Future randomized controlled trials are needed to confirm our findings and provide high-level evidence; however, the evidence currently available through our NMA encourages the usage of modern UCFB and MCFB insert designs.

## Figures and Tables

**Table 1:** Study characteristics.

Study	Group	Country	Implant Type	Patients	Patellar Resurfacing	Mean Age (SD/Range)	Sex (M/F)	Follow-Up (yrs)
Kim 2016 [20]	Ultracongruent FB	Korea	E-motion, Aesculap, B.Braun	82	Y	69.2	95% (4/78)	3
	Standard PS FB		E-motion, Aesculap, B.Braun					
Laskin 2000 [21]	Ultracongruent FB	USA	Genesis II, Smith and Nephew	128	Y	72.5	128 (NA)	2
	Standard PS FB		Genesis II, Smith and Nephew					
Lutzner 2016 [13]	Ultracongruent FB	Germany	Columbus, Aesculap, B.Braun	122	N	70.7	67% (85/42)	1
	Standard PS FB		Columbus, Aesculap, B.Braun	122				
Lutzner 2021 [22]	Ultracongruent FB	Germany	Columbus, Aesculap, B.Braun	122	N	71.5	68.2% (34/73)	5
	Standard PS FB		Columbus, Aesculap, B.Braun	122				
Uvehammer 2001 [23]	Ultracongruent FB	Sweden	AMK, DePuy, Johnson & Johnson	22	N	70.5	55% (22/27)	2
	Standard PS FB		AMK, DePuy, Johnson & Johnson					
Song 2017 [24]	Ultracongruent FB	Korea	E-motion, Aesculap, B.Braun	76	N	68.3	84% (12/64)	3.7
	Standard CR FB		E-motion, Aesculap, B.Braun					
Rajgopal 2017 [25]	Ultracongruent FB	India	Persona, Zimmer Biomet	105	N	NA	62% (39/66)	2
	Standard CR FB		Persona, Zimmer Biomet					
Indelli 2019 [26]	Medial Congruent FB	USA	Persona, Zimmer Biomet	100	Y	67.5	7% (93/7)	2
	Standard PS FB		Persona, Zimmer Biomet					
Kim 2009 [27]	Medial Congruent FB	KOREA	Wright Medical	92	Y	69.5	92% (7/85)	2.6
	Standard RP		PFC Sigma, DePuy, Johnson & Johnson					
Dowsey 2020 [28]	Medial Congruent FB	Australia	GMK, Medacta	75	Y	67.2	44% (46/36)	1
	Standard CR FB		GMK, Medacta					
	Standard PS FB		GMK, Medacta					
Kulshrestha 2020 [29]	Medial Congruent FB	India	ADVANCE, Wright	73	N	64.9	65% (28/52)	2
	Standard PS FB		NexGen Legacy, Zimmer					
Lee 2020 [30]	Medial Congruent FB	China	N/A	46	N	70	70% (14/32)	1
	Standard PS FB		N/A					
French 2020 [31]	Standard CR FB	Australia	Vanguard, Zimmer Biomet	90	N	67.8	58% (38/52)	1
	Medial Congruent FB		SAIPH, MatOrtho					
Edelstein 2020 [32]	Medial Congruent FB	United States	GMK, Medacta	50	Y	65.5	66% (17/33)	2
	Standard PS FB		GMK, Medacta					
Nishitani 2018 [33]	Medial Congruent FB	Japan	Bi-Surface, Kyocera	65	Y	74.1	74% (17/48)	2
	Ultracongruent FB (DH)		Bi-Surface, Kyocera					
Ishida 2012 [34]	Medial Congruent FB	Japan	ADVANCE, Wright	40	N	71.5	95% (2/38)	4.75
	Ultracongruent FB (DH)		ADVANCE, Wright					
Pritchett 2011 [16]	Medial Congruent FB	United States	Wright	440	Y	68	N/A	7.6
	Standard CR FB		Biomet/BioPro/DePuy/Stryker/WMT/Zimmer					
	Standard PS FB		Biomet/BioPro/DePuy/Stryker/WMT/Zimmer					
	Standard RP		DePuy, Johnson & Johnson					
Hossain 2011 [35]	Standard PS FB	UK	PFC DePuy, Johnson & Johnson	80	Y	70.7	66% (27/53)	2
	Medial Congruent FB		MRK; Finsbury Orthopaedics					
				1793				

## Data Availability

Not applicable.

## Appendix A. Mean Rank and SUCRA

A. Revision Rate

B. Radiolucent Lines

C. Range of Motion (ROM)

D. The Knee Society Score (KSS)

E. The Knee Society Score—Function (KSS-F)

F. Oxford Knee Score (OKS)

G. WOMAC

H. Summary of the functional outcomes, time to aseptic loosening, radiolucent lines, and revision by implant type

**Study**	**Group**	**ROM (Pre-op, Post-op, Change)**	**KSS (Pre-op, Post-op, Change)**	**KSS-F (Pre-op, Post-op, Change)**	**KSS-P (Pre-op, Post-op, Change)**	**OKS (Pre-op, Post-op, Change)**	**WOMAC (Pre-op, Post-op, Change)**	**Aseptic Loosening, Time**	**Radiolucency/Timing/Locations**	**Revision Rate/Time of Follow-Up/Reason**
Laskin 2000 [21]	Ultracongruent FB	112, 116, 4	36, 92, 56	50, 65, 15	18, 46, 28	N/A	N/A	N/A	N/A	0
	Standard PS FB	115, 116, 1	32, 94, 62	45, 55, 10	14, 44, 30	N/A	N/A	N/A	N/A	0
Lutzner 2017 [13]	Ultracongruent FB	N/A	36.6, 86.1, 49.5	42.5, 65.3, 22.8	N/A	19.5, 38.1, 18.6	N/A	N/A	N/A	1/9th month/deep infection
	Standard PS FB	N/A	81.6, 81.6, 0	47.3, 64.3, 17	N/A	20.8, 34.6, 13.8	N/A	N/A	N/A	1/3rd month/periprosthetic fracture after a fall
Kim 2016 [20]	Ultracongruent FB	119.1, 91.6, −27.5	47.3, 89.2, 41.9	37.5, 14.9, −22.6	N/A	N/A	52.9, 2.9. −50	0	0	N/A
	Standard PS FB	117.4, 94.3, −23.1	52, 90.3, 38.3	39.4, 14.5, −24.9	N/A	N/A	48.3. 2.4. −45.9	0	0	N/A
Lutzner 2021 [22]	Ultracongruent FB	N/A	N/A	N/A	N/A	20, 42, 22	N/A	1, 29th month after surgery	N/A	3/1 before 1-yr follow-up, and the other 2 before 3-yr follow-up/one of them had aseptic loosening (29th month), 22, 41, 19, the others had deep infection
	Standard PS FB	N/A	N/A	N/A	N/A	N/A	N/A	0	N/A	1/3rd month/medial condyle fracture
Uvehammer 2001 [23]	Ultracongruent FB	N/A	N/A	N/A	N/A	N/A	N/A	N/A	0	1/ N/A
	Standard PS FB	N/A	N/A	N/A	N/A	N/A	N/A	N/A	0	1/ N/A
Song 2017 [24]	Ultracongruent FB	116.3, 130.8, 14.5	N/A, 92.3, N/A	N/A	N/A	N/A	65.2, 25.2, −40	N/A	N/A	N/A
	Standard CR FB	116.1, 128.7, 12.6	N/A, 89.6, N/A	N/A	N/A	N/A	69.2, 24, −45.2	N/A	N/A	N/A
Rajgopal 2017 [25]	Ultracongruent FB	N/A	42.2, 72.8, 30.6	N/A	N/A	N/A	N/A, 19.45, N/A	N/A	N/A	0
	Standard CR FB	N/A	42.2, 79.7, 37.5	N/A	N/A	N/A	N/A, 15.6, N/A	N/A	N/A	0
Indelli 2019 [26]	Medial Congruent FB	112, 120, 8	64.4, 161.5, 97.1	N/A	N/A	19.6, 40.5, 20.9	N/A	0	2/2-yr follow-up/tibial	0
	Standard PS FB	108, 123, 15	63.7, 165.7, 102	N/A	N/A	19, 41.1, 22.1	N/A	0	3/2-yr follow-up/tibial	0
Kim 2009 [27]	Medial Congruent FB	N/A, 87, N/A	29, 87, 87	45, 80, 35	0, 35, 35	N/A	N/A	N/A	8/2.6-yr follow-up/2 in femoral, 6 in tibial (side)	0
	Standard RP	N/A, 94, N/A	28, 94, 66	45, 86, 41	0, 45, 45	N/A	N/A	N/A	5/2.6-yr follow-up/1 in femoral, 4 in tibial (side)	0
Dowsey 2020 [28]	Medial Congruent FB	N/A	29.1, 61, 31.9	29.4, 70.4, 41	N/A	16.2, 33.8, 17.6	59.6, 23, −36.6	N/A	N/A	N/A
	Standard CR FB	N/A	32.2, 60.8, 28.6	30.1, 64.7, 34.6	N/A	18.9, 33, 14.1	56.8, 28.3, −28.5	N/A	N/A	N/A
	Standard PS FB	N/A	33.5, 62.4, 28.9	29.3, 65.7, 36.4	N/A	17.8, 32.4, 14.6	57, 19.2, −37.8	N/A	N/A	N/A
Kulshrestha 2020 [29]	Medial Congruent FB	113.9, 107.8, −6.1	16.9, 44.5, 27.6	N/A	N/A	N/A	N/A	0	N/A	N/A
	Standard PS FB	108.8, 118.1, 9.3	17, 67.5, 50.5	N/A	N/A	N/A	N/A	0	N/A	N/A
Lee 2020 [30]	Medial Congruent FB	97, 108, 11	N/A, 91, N/A	57, 58, 1	21, 46, 27	N/A	49, 19, −30	N/A	N/A	N/A
	Standard PS FB	100, 110, 10	N/A, 90, N/A	49, 60, 11	17, 47, 30	N/A	49, 16, −33	N/A	N/A	N/A
French 2020 [31]	Standard CR FB	104.9, 114.3, 9.4	N/A	N/A	N/A	20.2, 41, 20.8	50.5, 11.4, −39.1	N/A	N/A	N/A
	Medial Congruent FB	102.7, 115.1, 12.4	N/A	N/A	N/A	20.5, 42.2, 21.7	50.5, 8.6, −41.9	N/A	N/A	N/A
Edelstein 2020 [32]	Medial Congruent FB	N/A	N/A, 86, N/A	N/A, 77.4, N/A	N/A	19.88, 38.28, 8.4	N/A	N/A	N/A	0
	Standard PS FB	N/A	N/A, 88.1, N/A	N/A, 81.4, N/A	N/A	16.31, 40.41, 24.1	N/A	N/A	N/A	0
Nishitani 2018 [33]	Medial Congruent FB	98.8, 106.4, 7.6	39.1, 76.5, 37.4	44.2, 51.9, 7.7	N/A	N/A	N/A	N/A	N/A	0
	Ultracongruent FB (DH)	98.1, 103.2, 5.1	37.4, 70.5, 33.1	42.5, 51.2, 8.7	N/A	N/A	N/A	N/A	N/A	0
Ishida 2012 [34]	Medial Congruent FB	110, 110, 0	34, 89, 55	40, 65, 25	10, 45, 35	N/A	N/A	N/A	0	0
	Ultracongruent FB (DH)	110, 115, 5	36, 85, 49	45, 65, 20	10, 45, 35	N/A	N/A	N/A	0	0
Pritchett 2011 [16]	Medial Congruent FB	N/A	N/A, 93, N/A,	N/A, 80.4, N/A	N/A	N/A	N/A	N/A	0	N/A
	Standard CR FB	N/A	N/A, 90, N/A	N/A, 71.3, N/A	N/A	N/A	N/A	N/A	0	N/A
	Standard PS FB	N/A	N/A, 92, N/A	N/A, 74.1, N/A	N/A	N/A	N/A	N/A	0	N/A
	Standard RP	N/A	N/A, 90, N/A,	N/A, 81.1, N/A	N/A	N/A	N/A	N/A	0	N/A
Hossain 2010 [45]	Standard PS FB	93.9, 100.1, 6.2	48.4, 68.6, 20.2	47.9, 68, 20.1	N/A	41.7, 29.1, −12.6	53.8, 32.9. −20.9	N/A	0	0
	Medial Congruent FB	97.3, 114.9, 17.6	43, 76.3, 33.3	44.6, 71.4, 26.8	N/A	41.6, 26.2, −15.4	56. 17.3. −28.9	N/A	0	0
